# *EGFR*和*ALK*基因异时性突变非小细胞肺癌1例报告并文献复习

**DOI:** 10.3779/j.issn.1009-3419.2024.106.15

**Published:** 2024-07-20

**Authors:** Xiaoyan KONG, Mingjuan WANG, Qiaoyun TANG, Mengyu SUN, Jianjun HU

**Affiliations:** 211102 南京，南京同仁医院肿瘤科; Department of Oncology, Nanjing Tongren Hospital, Nanjing 211102, China

**Keywords:** 异时性多原发肺癌, 肺内转移, EGFR, ALK, Metachronous multiple primary lung cancer, Intrapulmonary metastasis, EGFR, ALK

## Abstract

多原发肺癌（multiple primary lung cancer, MPLC）指患者有两个或两个以上原发病灶的肺癌，根据发生时间的不同分为同时性多原发肺癌（synchronous MPLC, sMPLC）和异时性多原发肺癌（metachronous MPLC, mMPLC）。近年来，MPLC的检出率逐渐升高，但由于肿瘤的异质性，在鉴别MPLC和肺内转移（intrapulmonary metastasis, IM）上存在许多争议，特别是病理组织学类型相同时。考虑到目前二者在临床治疗策略及预后上的显著差异，对于MPLC和IM的精确诊断是个体化精准治疗的关键。分子遗传学及测序技术为检测肿瘤的克隆性起源提供了有效的策略，其中非小细胞肺癌表皮生长因子受体（epidermal growth factor receptor, EGFR）突变与间变性淋巴瘤激酶（anaplastic lymphoma kinase, ALK）融合突变共存的病例陆续有报道，但ALK基因突变后再发EGFR突变的案例未见提及。本文通过分子遗传学技术准确诊断并回顾性分析了1例ALK突变型男性肺腺癌患者术后4年再发EGFR突变合并多发转移的临床资料，并复习相关文献，以期加深对mMPLC的认识，为该类病例的诊疗提供临床借鉴。

## Abstract (en)

Multiple primary lung cancer (MPLC) refers to patients with two or more primary lesions of lung cancer. It can be divided into synchronous MPLC (sMPLC) and metachronous MPLC (mMPLC) based on the timing of occurrence. In recent years, the detection rate of MPLC has gradually increased. However, considerable controversy exists in distinguishing MPLC from intrapulmonary metastasis (IM), especially when the histopathological types are identical. Given the significant differences in treatment strategies and prognosis in clinical practice currently, accurate diagnosis of MPLC is crucial for personalized precision therapy. Molecular genetics and sequencing technologies offer effective strategies for assessing the clonal origin of tumors. There have been reports of coexisting mutations in the epidermal growth factor receptor (EGFR) and anaplastic lymphoma kinase (ALK) fusion genes in non-small cell lung cancer, but case of EGFR mutation following an ALK mutation has not been mentioned. This article accurately diagnoses and retrospectively analyzes the clinical data of a case of ALK mutant adenocarcinoma in a male patient who developed an EGFR mutation with multiple metastases four years after surgery, and reviews the relevant literature. This paper aims to deepen the understanding of mMPLC and provide clinical references for the diagnosis and treatment of such patients.

肺癌的发病率及死亡率高居全球癌症前列，但随着对肺癌致病机制研究的深入，非小细胞肺癌（non-small cell lung cancer, NSCLC）中的驱动基因不断被揭示，如表皮生长因子受体（epidermal growth factor receptor, EGFR）、间变性淋巴瘤激酶（anaplastic lymphoma kinase, ALK）、Kirsten鼠肉瘤基因（Kirsten rat sarcoma viral oncogene, KRAS）以及间充质细胞上皮转化因子（mesenchymal-epithelial transition factor, MET）等，这些驱动基因的发现为后续的靶向治疗提供了分子靶点^[1]^。尤其是靶向EGFR和ALK抑制剂的应用，在一定程度上改善了患者的生存质量，延长了患者的生存期。既往研究^[2]^发现，在NSCLC中ALK重排与EGFR外显子缺失突变是相互排斥的分子事件。然而随着分子遗传学和测序技术在癌症检测中的应用，EGFR和ALK基因双突变共存的NSCLC研究^[3,4]^陆续有报道，常多见于同时性多原发肺癌（synchronous multiple primary lung cancer, sMPLC），但是ALK突变后再发EGFR突变的异时性多原发肺癌（metachronous MPLC, mMPLC）案例未见报道。本文在获得患者知情同意下，将2021年9月南京同仁医院收治的1例ALK突变型术后4年再发EGFR突变合并多发转移的晚期男性肺腺癌病例的临床资料报道如下，旨在为mMPLC患者的诊断和治疗提供借鉴。

## 1 病例资料

患者，男，66岁，因“右肺腺癌IIIA期术后4年8月，胸闷气喘6月、加重1天”于2022年3月19日就诊于南京同仁医院。既往“2型糖尿病”病史8年余，现“门冬胰岛素30早9 iu、晚7 iu”皮下注射，血糖控制良好。否认吸烟史。其父亲、1个哥哥、1个姑姑有肺癌病史，具体不详。患者2017年6月胸部电子计算机断层扫描（computed tomography, CT）示右上肺占位，2017年7月6日在南京鼓楼医院行正电子发射型计算机断层显像（positron emission tomography, PET）/CT显示，右肺上叶软组织结节伴瘤周结节，代谢局限性增高，考虑肺癌可能性大。2017年7月18日于外院行“右肺上叶占位切除术”，术后病理为右肺上叶肺腺癌，腺泡为主型（腺泡型约70%，乳头型约20%，贴壁型约10%），大小约2.3 cm×2.0 cm×1.4 cm，上纵隔淋巴结查见淋巴结1/2枚见癌组织转移；免疫组化为肿瘤细胞表达人表皮生长因子受体2（human epidermal growth factor receptor 2, HER2）（0），EGFR（+），细胞程序性死亡配体1（programmed cell death ligand 1, PD-L1）（-），肿瘤增殖标记物Ki-67（marker of proliferation Ki-67, Ki-67）（约5%+）；VENTANA anti-ALK（D5F3）肿瘤细胞：（+++）（图1A）；对照组织：阳性对照（+），阴性对照（-）。术后分期IIIA（pT1cN2M0）期，术后未行抗肿瘤治疗，定期复查未见复发转移。2021年8月31日患者因胸闷、气喘曾行胸部CT检查，未明确诊断复发转移，诊断为慢性阻塞性肺疾病急性加重期，予止咳化痰等对症处理后患者咳嗽稍好转，胸闷、气喘症状未见改善。2022年3月19日患者因胸闷气喘进行性加重、呼吸困难至南京同仁医院急诊就诊，查胸部CT平扫显示：右肺癌术后复查；右肺不张，较前片（2021年8月31日）新发；左肺粟粒样结节，较前新发，考虑癌性淋巴管炎可能；纵隔及右侧腋窝稍大淋巴结，较前右侧腋窝稍增大；心影增大，心包积液，较前新发；双侧胸膜转移可能；左侧叶间裂及胸腔积液，较前新发；T_1_椎体高密度影，较前相仿，转移待排；上腹部脂肪间隙模糊伴结节影，转移可能，较前新发。2022年3月20日查糖类抗原125为175.0 U/mL。2022年3月21日胸腹部增强CT（图1B、1C）：右肺不张，左肺粟粒样结节，考虑癌性淋巴管炎；纵隔及右侧腋窝稍大淋巴结；双侧胸膜转移可能；肝脏多发转移可能。支气管镜检查：（右肺下叶）：黏膜固有层见异体腺样结构，结合病史，考虑肺腺癌。免疫组化：甲状腺转录因子-1（thyroid transcription factor-1, TTF1）（+++）。患者肺癌IV期诊断明确，发病急，胸闷气喘明显，急需控制肿瘤、改善症状。因患者既往术后ALK阳性，给予阿来替尼600 mg bid口服1周，复查胸部CT（图1D）较前相仿，服药期间患者胸闷气喘无改善。2022年3月31日基因检测（表1）报告：EGFR exon19 del阳性。2022年4月1日开始阿美替尼110 mg qd口服2周，后患者胸闷、气喘症状改善。2022年4月15日复查胸部CT（图1E）：右肺不张及左肺粟粒样结节好转，纵隔及右侧腋窝稍大淋巴结缩小。根据实体肿瘤疗效评价标准（Response Evaluation Criteria in Solid Tumors, RECIST）1.1版，疗效为部分缓解。继续阿美替尼110 mg qd口服，并于2022年4月18日、2022年5月16日联合培美曲塞二钠0.7 g静滴+卡铂400 mg静滴q3w治疗2个周期。2022年6月7日腹部增强CT（图1F）示肝脏部分异常强化灶未见显示。因患者出现2级乏力、食欲下降及骨髓抑制，耐受不佳，卡铂减量处理。2022年7月15日行培美曲塞二钠0.7 g静滴+卡铂200 mg静滴治疗1个周期，患者仍出现2级乏力，后停用卡铂，培美曲塞二钠减量处理。2022年8月8日开始行阿美替尼110 mg qd口服+培美曲塞二钠0.6 g静滴q3w维持治疗，期间多次影像学评估病情稳定，末次影像学检查为2024年2月27日（图1G、1H）。截至2024年4月患者无进展生存期（progression-free survival, PFS）已达24个月。

**图1:**
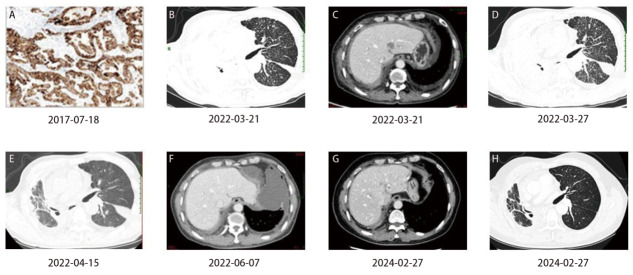
患者病理结果及影像学表现。A：患者肺癌术后免疫组织化学显示ALK（D5F3）呈阳性表达（VENTANA法，×200）；B：病情进展时基线胸部CT示左肺粟粒样结节、癌性淋巴管炎、右肺不张；C：病情进展时基线腹部CT示肝转移；D：阿来替尼靶向治疗1周后复查胸部CT示较基线相仿，无改善；E：阿美替尼靶向治疗2周后复查胸部CT示右肺不张及左肺粟粒样结节好转；F：阿美替尼靶向治疗2个月后复查腹部CT示肝部分转移灶消失；G：阿美替尼靶向治疗22个月后腹部CT提示腹部病情稳定；H：阿美替尼靶向治疗22个月后胸部CT提示胸部病情稳定。

**表1:** 患者基因检测结果

Gene name	Exon	Nucleotide variation	Result	Medication tips
EGFR	18	p.G719A/S/C	Negative	
	19	Del	Positive	
	20	Ins, p.S7681, p.T790M	Negative	Gefitinib
	21	p.L858R, p.L861Q	Negative	Erlotinib
ERBB2	20	p.Q61R/K/L/H	Negative	Icotinib
KRAS	2	-	Negative	Afatinib
BRAF	15	p.V600E/K/E2/R/D1/D2	Negative	Dacomitinib
ALK fusion	-	-	Negative	Osimertinib
ROS1 fusion	-	-	Negative	Almonertinib
RET fusion	-	-	Negative	
MET	14-skipping	-	Negative	

EGFR: epidermal growth factor receptor; ERBB2: Erb-B2 receptor tyrosine kinase 2; KRAS: Kirsten rat sarcoma viral oncogene; BRAF: V-raf murine sarcoma viral oncogene homolog B; ALK: anaplastic lymphoma kinase; ROS1: ROS proto-oncogene 1; RET: rearranged during transfection; MET: mesenchymal-epithelial transition factor; Del: delete; Ins: insert; -: Exon or nucleotide mutation sites and fusion genes are too many to list.

## 2 讨论

关于MPLC的诊断，早在2003年美国胸科医师协会（American College of Chest Physicians, ACCP）肺癌指南中已经提出相关诊断标准，且多次做出更新^[5]^，具体参考以下三点：（1）具有相同的组织学类型且解剖上不相连：位于不同肺叶、没有N2或N3淋巴结转移、没有全身转移；（2）具有相同的组织学类型，无瘤间期≥4年、两个癌灶都没有全身转移；（3）不同的组织学类型，或不同的基因突变，或分别起源于原位癌灶。除此之外，对于MPLC诊断还增加了分子遗传学的分析，并强调了学科间的交叉与联合在诊断过程中的重要性。特别是当mMPLC的不同癌灶表现为相同的组织学类型，如肺腺癌，很难有效辨别mMPLC和肺内转移（intrapulmonary metastasis, IM），而且由于二者的诊疗和预后存在显著差异^[6]^，对于MPLC与IM的准确区分尤为重要。

组织病理学是诊断的金标准。组织病理学的综合评估（comprehensive histologic assessment, CHA）也是鉴别MPLC和IM主要方法，旨在通过比较不同病灶间组织学类型、主要组织学亚型、次要组织学亚型、细胞学和基质特征（是否有坏死、炎症、淋巴增生和角化等）等实现MPLC和IM的精确鉴别，而且CHA的结果与分子评估结果也高度吻合^[7]^。但是，临床上绝大多数多原发肿瘤的组织学类型是极为相似的。例如该患者初诊、再次确诊的组织学类型均为腺癌，且再次发病后伴有多部位的肿瘤转移，并不符合MPLC的诊断标准。因此，通过组织病理学鉴别依然存在很大的困难和局限性。

影像学检查是肺癌筛查最有效的方式之一，胸部CT或PET/CT在一定程度上可有效区分MLPC和IM。临床上主要依据不同病灶的形态和PET/CT的标准化摄取值（standardized uptake value, SUV）等指标综合诊断，通常在CT影像上，MPLC以磨玻璃成分多见，IM则以实性成分为主且边缘光滑，少见毛刺或分叶征^[8]^。另有多项研究^[9,10]^也表明，不同患者肿瘤多个病灶的SUV差异值（difference between SUVs, ∆SUV）显著，特别是MPLC患者的∆SUV明显高于IM患者。但是，这一结论仍然需要更多数据的支持；而且，基于影像学指标诊断MPLC和IM主观性较强，缺乏统一的标准。此外，面对患者已经多发转移的情况，这种鉴别存在很大的局限性。

分子遗传学和测序技术发展为MPLC诊断提供了新的策略，特别是对于组织病理学评估和影像学检测难以有效进行鉴别的肿瘤类型。2013年ACCP指南提出通过分子遗传学特征鉴别多发肺癌的克隆性起源^[11]^。目前基因组测序、外显子测序以及下一代测序等技术已被运用于MPLC的特征分析，筛选出EGFR、ALK、KRAS、c-ros肉瘤致癌因子-受体酪氨酸激酶（ROS proto-oncogene 1-receptor tyrosine kinase, ROS1）、MET、鼠类肉瘤滤过性毒菌致癌同源体B1基因（V-Raf murine sarcoma viral oncogene homolog B1, BRAF）、表皮生长因子受体2（Erb-B2 receptor tyrosine kinase 2, ERBB2）和转染重排基因（rearranged during transfection, RET）等一系列驱动基因^[12⇓⇓-15]^。研究^[16,17]^发现，NSCLC中EGFR突变和ALK重排是肺腺癌最为主要的驱动基因，EGFR突变和ALK重排导致下游通路的持续激活，诱发细胞异常增殖和侵袭使得正常细胞癌变。此外，对于驱动基因突变频率的分析有助于MPLC的鉴别，即若多个癌灶存在同一驱动基因突变，该驱动基因突变频率越低，IM的可能性越大；反之，对于具有相同高频驱动基因突变的患者，在诊断时需要考虑MPLC的可能^[18]^。近年来深度学习及人工智能的发展，使其在肿瘤影像及病理等领域有了诸多应用^[19,20]^。虽然目前相关研究鲜有报道，但人工智能必将为MPLC的诊断带来新的解决方案。

本例患者初诊肺癌时术后病理ALK阳性，4年余后病情进展，且伴有多部位的肿瘤转移，从影像学及组织病理学上分析并不符合MPLC的诊断标准，故按照转移性肺癌去治疗，但效果不佳，给初期的诊断和治疗带来了困难，也反映了目前的诊断标准在复杂的临床实践下还是存在很多不足，后又进行驱动基因检测，提示EGFR exon19 del阳性，由此明确该患者是mMPLC。根据2022中国临床肿瘤学会指南，IV期敏感基因突变NSCLC一线治疗首选厄洛替尼、吉非替尼、埃克替尼、达可替尼、阿法替尼、奥希替尼、阿美替尼、伏美替尼^[21]^。FLAURA研究^[22]^后，由于奥希替尼改善了患者的总生存期，三代酪氨酸激酶抑制剂（tyrosine kinase inhibitors, TKIs）已经成为了肺癌敏感基因突变患者的一线治疗优选方案。AENEAS研究^[23]^是一项评估中国患者EGFR敏感基因突变、阿美替尼作为一线治疗的多中心、随机、双盲III期研究，相较于吉非替尼，该研究取得了中位PFS 19.3 vs 9.9个月的结果，显示了很好的疗效。此外，NCT04646824研究^[24]^表明，阿美替尼+培美曲塞+卡铂一线治疗EGFR敏感突变NSCLC患者展示出更好的客观缓解率和疾病控制率，考虑患者肿瘤负荷大，合并胸闷、气喘等症状，急需快速缩瘤减症，故一线治疗选择阿美替尼联合化疗，治疗期间通过药物对肿瘤的快速控制，胸闷、气喘等症状快速缓解，患者生活质量明显改善。此外，治疗期间应根据患者不良反应、耐受性、疗效等，个体化调整药物剂量、周期等，使患者获益最大化。

MPLC在临床相对少见，易误诊或漏诊。近年来MPLC和IM的鉴别诊断取得了快速发展，越来越多的研究者开始认同将分子生物学方法应用于MPLC的诊断及鉴别诊断。MPLC和IM预后存在显著差异，这提醒我们在临床工作中，不仅要提高对肺癌的诊断，也要高度警惕肺癌复发的诊断，主动进行mMPLC的鉴别，同时还要关注再发其他类型原发肺癌的鉴别诊断，不断加深对MPLC的认识。对于不同驱动基因突变在多原发肿瘤基础上出现了转移，应及时修正诊疗方案，改善患者生存。随着对MPLC临床和基础研究的不断深入，多学科参与的规范化和个体化诊治将是未来的主要发展方向。
